# Experiences of adolescents and parents on the mental health management of depression in adolescents, North West province, South Africa

**DOI:** 10.4102/curationis.v45i1.2178

**Published:** 2022-02-16

**Authors:** Precious C. Chukwuere, Leepile A. Sehularo, Mofatiki E. Manyedi

**Affiliations:** 1 Department of Nursing Science, Faculty of Health Science, North-West University, Mahikeng, South Africa

**Keywords:** adolescents, depression, experience, management, North West province, parents, South Africa

## Abstract

**Background:**

Living with or managing an adolescent suffering from depression predisposes the adolescent and parents to various experiences, considering the multifactorial nature of depression and associated symptoms.

**Objective:**

This study explored and described the experiences of adolescents and their parents on the mental health management of depression in the North West province (NWP), South Africa.

**Method:**

A qualitative, exploratory, descriptive, contextual research design was adopted. Data was collect from two mental health care institutions and two mental health care units attached to two general hospitals in the NWP, SA. Thirty-two participants (18 adolescents and 14 parents) were purposefully selected for the study. Data were collected through individual interviews and analysed using Tesch’s open-coding method to generate themes and categories which were presented with the concurrent support of participants direct quotations.

**Results:**

The study revealed that the experiences of adolescents with depression and their parents taking care of them at homes include the following: emotional distress, poor coping mechanisms, financial burden, repeated suicidal attempts, negative attitudes from support systems and withdrawal behaviours. Appropriate therapeutic environments, ongoing monitoring by mental healthcare practitioners and adequate support systems were suggested by participants as management approaches that could enhance the recovery of adolescents from depression.

**Conclusion:**

The findings revealed the devastating experiences of adolescents with depression and their parents taking care of them in their various homes which confirms the dire need for attention on the plights of these groups in order to facilitate adolescents’ recovery and strengthen the adolescents’ and parents’ coping mechanisms for a healthier family.

## Introduction

Adolescence is characterised by different physical, biological and emotional changes, predisposing the individual to mental health disorders, such as depression (Stikkelbroek et al. 2016:2). It is estimated that depression affects 8% – 21% of adolescents before the age of 18 years, with devastating consequences (Davies, Sullivan & Zammit 2018:497). Depression in adolescence can be because of interpersonal stress (Deo & Prelow 2018:2), gender-associated challenges (Nalugya-Sserunjogi et al. 2016:2), parental depression (Thapar et al. 2012:1056), difficulty in emotional regulation, relationship break-ups or bullying from peers (Chukwuere, Pienaar & Sehularo 2020a:125; Stikkelbroek et al. 2016:2). Symptoms of depression in adolescents include loss of interest in pleasurable activities, sadness, low mood and increased fatigability (Chukwuere, Pienaar & Sehularo 2020b:15 090; Wartberg, Kriston & Thomasius 2018:549). These symptoms contribute to the devastating experience of the adolescents with depression, heightening its consequences. Stikkelbroek et al. (2016:2) consider comorbid psychiatric disorders, high reoccurrence tendency and suicidal risks as some of the consequences of depression amongst this age group. McLeod, Horwood and Fergusson (2016:1401) further maintained that depression amongst adolescents is associated with poor social functioning, poor academic performance, poor self-esteem and unemployment.

Parents of adolescents suffering from depression play crucial roles in the management of depression, coupled with the personal factors of such adolescents (Nelis et al. 2019:257). Parental involvement in the management of adolescent depression facilitates adolescent adherence to treatment guidelines, thereby improving their treatment outcomes (Dardas, Van de Water & Simmons 2018:567). Mental health managements also contributes to the amelioration of symptoms of depression in adolescents (Cheung et al. 2018:11). These managements include the treatments prescribed by the mental healthcare practitioners, the activities of the adolescents and parents focused on facilitating the recovery of adolescents from depression. However, management of depression exposes adolescents and their parents to different experiences, such as feeling overwhelmed or traumatised, caused by the symptoms, consequences and effects of managing the mental health condition (Radovic et al. 2015:5). Adolescents suffering from depression live in fear of potentially falling back to depression, despite the ongoing management (Kuwabara et al. 2007:317). Such experiences interfere with their sense of identity as such experiences are contrary to how adolescents receiving management for depression should perceive themselves (De Mol, D’Alcantara & Cresti 2018:2). Thus, adolescents suffering from depression appear to self-alienate, despite the mental health management, which also negatively affects their parents, family members and friends (De Mol et al. 2018:2).

Studies on depression in adolescents have mainly focused on aetiology of depression (Stikkelbroek et al. 2016:2), prevalence of depression (Davies et al. 2018:497) and psychosocial management of depression amongst adolescents (Chukwuere et al. 2020a:125). However, Stapley, Midgley and Target (2016:619) observed that despite the increased prevalence of depression amongst adolescents and the associated scholarly works, a very few studies have focused on the experiences of adolescents and their parents on the management of depression. Thus, the experiences of adolescents suffering from depression and that of the parents managing them at homes should be an area of great concern (Stapley et al. 2016:618).

Despite the literature on depression and its prevalence amongst adolescents, to the best of our knowledge, no overtly published studies have focused on the experiences of adolescents and their parents on mental health management received by the depressed adolescents in the North West province (NWP), South Africa. Thus, a qualitative study on the experiences of adolescents and their parents on the mental health management of depression will help to unpack responses to the management of depression and address gaps in the literature. The aim of this study was, therefore, to explore and describe the experiences of adolescents and parents on the mental health management received by adolescents with depression in the NWP, South Africa. Parental involvement in this study is crucial to facilitate the comprehensive understanding of the plights of living with and managing adolescents with depression.

## Research methodology

### Research design

A qualitative, exploratory, descriptive, contextual research design was used to explore and describe the experiences of adolescents and their parents on the mental health management of depression in the NWP, South Africa (Polit & Beck 2017:98). This research design allowed for in-depth exploration of the experience of adolescents and parents on the mental health management of depression, thereby, enabling the generation of quality data for analysis. Thus, a qualitative, exploratory, descriptive, contextual research design was considered the most appropriate for this study.

### Study context

This study was conducted in outpatient departments of district hospitals in the NWP, namely, in Ngaka Modiri Molema, Dr Kenneth Kaunda, Dr Ruth Segomotsi Mompati and Bojanala districts. There is one mental healthcare institution in Ngaka Modiri Molema and one in Dr Kenneth Kaunda. There is one mental healthcare unit attached to the general hospital in Dr Ruth Segomotsi Mompati and one attached to the general hospital in Bojanala district.

### Population and sampling

The target population consisted of adolescents discharged three weeks after being diagnosed and admitted with depression and their parents, accompanying them for follow-up treatment in the outpatient departments of the two mental healthcare institutions and two mental healthcare units within the two general hospitals, NWP, South Africa. A non-probability, purposive sampling technique was used to select participants (adolescents and parents) for the study. The adolescents and their parents were selected because they could respond to the research questions. This sampling technique was considered appropriate for the study because it allowed the researcher to purposefully select participants based on their ability and positions to appropriately answer the research questions. In addition, purposive sampling enabled the researcher to recruit participants who were able to voice their opinions, experiences and views about the study.

Adolescents aged 14–17 years (boys and girls) were included in the study. These groups of adolescents were considered appropriate by the researcher to answer the research questions and generate quality data. In addition, adolescents within this age bracket fall within the category of those as defined by the Demographic Profile of Adolescents (DPA) in South Africa. Furthermore, adolescents who were in the post-discharge period (three weeks) after being diagnosed with depression were considered for the study. These adolescents were not undergoing active treatment for depression, and thus, chances of triggering symptoms were minimal, and they were able to respond to the research questions. This group comprised of adolescents who were non-psychotic but undergoing treatment in mental healthcare institutions or units for depression. Non-psychotic but depressed adolescents had better chances of providing answers to the research questions. In addition, participants who could understand and speak English or Setswana were selected.

Parents of adolescents undergoing treatment for depression and those visiting the outpatient units of mental healthcare institutions or mental healthcare units for check-ups were selected for the study. They were considered in the study based on their parenting roles and the primary care provided to adolescents. Parents are responsible for the following: they are psychological guardians; they provide food, clothing and shelter; behavioural nurturing; and spiritual guidance. Adolescents suffering from any form of psychotic disorder, neurological impairment or speech impairment that could prevent them from answering the research questions were excluded from the study. Actively suicidal adolescents were also excluded from the study.

Professional nurses (mediators) in the outpatient units of the two mental healthcare institutions and two mental healthcare units attached to the two general hospitals assisted in the initial recruitment of adolescents and their parents. The mediators identified adolescents as they visited the study areas with their parents for a check-up, informed parents about the study, sought their permission to approach adolescents, approached each adolescent without coercion, explained the study to them, conducted a mental assessment on adolescents, gave the contact details of the independent person (negotiator), for those interested to make appointments for the signing of permission, and informed consent forms. The mediator also gave copies of the various consent forms to the adolescents and their parents to read at home before making an informed decision.

The mediator further called the researcher and the negotiator and informed them to meet the adolescents and their parents who showed interest in further recruitment processes. The meeting was to ensure that the mediator does not serve as the main participant recruiter and to avoid issues associated with hierarchical relationships. The mental assessment of adolescents was to ascertain whether they were psychotic as psychotic adolescents were considered not eligible for the study. The professional nurses also assisted with the initial recruitment of adolescents and their parents because of their employment within the outpatient unit, and the fact that they had a better understanding of the group of participants selected for the study (Tracy 2019:17). Considering the nurse–patient relationship, the professional nurse politely approached adolescents and their parents regarding the study.

The negotiator was a full-time postgraduate student from the School of Nursing Science, Department of Health Sciences, North-West University, Mafikeng Campus. The duties of the negotiator were to facilitate further recruitment of participants (explanation of the study to the adolescents and their parents and allow them to make informed decisions) and signing of permission and consent forms. The mediators explained the purpose of the study to participants, such as data collection, venue for data collection, risks and benefits. All participants were provided enough time to make informed decisions before taking part in the study. The data were collected after the participants signed the consent forms. All expenses incurred by participants before their participation in the study, such as airtime to make calls, were reimbursed.

The research study involved a translator for data collection who also signed the confidentiality agreement form. The translator was a full-time postgraduate student from the Setswana Department in the North-West University, Mafikeng Campus, fluent in Setswana. The researcher explained the study to the translator and his duties, which include assisting during data collection and transcribing of the data. The translator was only involved in one of the interviews with a parent. The data from the parent were carefully transcribed by the translator and discussed with the researcher for confirmation of the data. The participant who required a translator was identified during the signing of permission and voluntary informed consent forms, hence the involvement of a translator. The translator accompanied the researcher to the study setting on the scheduled date for data collection and was involved to ensure there is adequate gathering of data from the participant.

### Data collection

Semi-structured face-to-face interviews were used to collect data from 32 participants (18 adolescents and 14 parents). Creswell (2014:234) states that an interview is usually carried out in the participant’s natural setting and is an interaction between the researcher and the participant(s) with the sole aim of asking questions that cover the research topic. A semi-structured interview enabled new ideas during the interview session through facilitating probing of the research question. Face-to-face individual interviews enabled the researcher to explore the research questions to better understand the experiences of adolescents and their parents on the mental health management of depression in adolescents, NWP, South Africa hence, best for the study. Each face-to-face individual interview section lasted 25–50 min depending on the willingness of the participants on whether to respond to some questions and data saturation. The researcher endeavoured to write field notes, which helped in capturing all the information from the participants regarding the study. The principle of data saturation was adhered to in this study. Data saturation refers to the point at which participants or groups of participants at any level of data collection are unable to put forth new information on the research questions (Creswell 2014:296). In addition, Ary et al. (2014:382) argued that data saturation is a point in qualitative data collection where participants no longer produce new information regarding the research questions. The main question asked was ‘what are the experiences of adolescents and parents on the mental health management of depression?’

### Data analysis

Data were analysed (separately) using Tesch’s open-coding method by the researcher and an independent co-coder, and a meeting was arranged to agree on the themes and categories (Creswell 2014:246). The steps for data analysis include transcribing the raw data verbatim, scanning through the raw data, carefully reading the transcribed data to have a general sense of the data and be able to reflect on their meanings, removing irrelevant details and preparing for coding the data, and generating and organising the concepts, categories and themes properly for clarity and validation. This was performed to ensure that that the data collected reflect the authentic ideas of participants (Creswell 2014:247).

**TABLE 1:** Themes and categories that emerged from the analysis.

Themes	Categories
Negative experiences with regard to depression amongst adolescents	Feelings of hopelessness and suicidal ideation
	Difficulty in coping
	Negative attitudes from the support system
	Financial strain
	School-related challenges
	Withdrawal
Methods of managing depression in adolescents	Provision of a therapeutic environment in hospitals
	Continuous monitoring by mental healthcare practitioners
	Positive attitude from support systems
	Use of media to teach people about depression

The themes and categories identified in the study are discussed in the following subsection, supported with direct quotations and the literature.

### Ethical considerations

Ethical approval to conduct the study was obtained from the Scientific Committee of the School of Nursing Science and the North-West University Health Research Ethics Committee (HREC Reference Number: NWU-00448-19-A1). Permission to conduct the study was requested and obtained from the North-West Provincial Department of Health, Chief Executive Officers of mental healthcare institutions selected for the study and the Chief Executive Officer of the two mental healthcare units within the two general hospitals. Official permission was also requested and obtained from parents of adolescents to participate in the study. The participants signed informed consent forms before participating in the study.

### Trustworthiness

Trustworthiness was ensured through maintaining credibility, confirmability, transferability and dependability, as outlined by Creswell (2014:251). Credibility was ensured by establishing a trusting relationship with the parents and adolescents and using an independent co-coder during data analysis (Polit & Beck 2017:241). Confirmability was ensured through objectivity during data collection and subsequent analysis (Creswell 2014:251). Objectivity was enhanced through the use of an independent co-coder and the promoters of the study acted as independent checkers throughout the research process. Transferability was maintained through a broad explanation of the study area, population, sampling techniques, data collection process, data analysis technique and discussion to have adequate information for the audience to make informed judgements regarding transferability (Polit & Beck 2017:246). Dependability was ensured through clear presentation of the research methodology and findings, thus fostering transparency of the study.

## Findings and discussion

### Demographic characteristics of participants

This study was conducted in two mental healthcare institutions and two mental healthcare units within the two general hospitals in the NWP, South Africa. Eighteen adolescents (14 females and 4 males) and 14 parents (13 females and 1 male) were recruited by a mediator. Adolescents selected for the study were young people visiting the two mental health institutions and two mental health units attached to the two general hospitals (aged 14–17 years) after being diagnosed with depression, whilst the parents were adults accompanying their children to these facilities for consultation. The adolescents and parents shared their experiences on the mental health management of depression in adolescents. The data collected from these participants were analysed with the generated themes and categories reflecting the views of the participants. The generated themes and categories reflect the views of the participants and were presented with the concurrent support of adolescents and parents’ direct quotations.

#### Theme 1: Negative experiences with regard to depression among adolescents

The following categories emerged from the negative experiences with regard to depression amongst adolescents: feeling of hopelessness and suicidal ideation, difficulty in coping, negative attitudes from support systems, financial strains, school-related challenges and withdrawal.

**Feelings of hopelessness and suicidal ideation:** Adolescents and their parents maintaining feelings of hopelessness and thoughts of committing suicide were examples of negative experiences with regard to the mental health management being received adolescents with depression. These experiences can make things more challenging to an adolescent suffering from depression and people around the individual whilst still on treatment as indicated in the following excerpts by participants:

‘I tried committing suicide because I feel hopeless, I was losing my mind and I feel killing myself will bring to an end all the suffering in this world is not interesting at all.’ (Adolescent 1, female, MHI)

‘I kept keeping my deep thoughts to myself, mood swing, too much of sleeping, feeling helpless and hopeless and they are not going away at all, it is not helping me in any way because I do not see the life I am living.’ (Adolescent 3, female, MHU)

‘I only realised when she attempted the first suicide, this time when she went to consult a psychologist, she told the psychologist how she attempted to kill herself and I was so shocked what if she killed herself, is the treatment not working?’ (Parent 5, female, MHU)

Depression aggravates and predisposes adolescents to hopelessness, helplessness and lack of interest in pleasure (Stikkelbroek et al. 2016:3). Neufeld et al. (2017:120) stated that adolescents suffering from depression experience suicidal thoughts and possible reoccurrence. Theule et al. (2013:5) posited that depression in adolescents increases stress on parents and families compared with parents or families without adolescents with depression. Huang et al. (2014:255) also affirmed that depression amongst this age group predisposes the family to physical and psychological stress.

**Difficulty in coping:** Adolescents and their parents indicated that managing depression can be challenging because of recurrent episodes and other associated consequences, thus contributing to the devastating experience of adolescents and those around them. Adolescents also revealed that despite mental health management strategies, they still find it difficult to cope with depression as it affects their daily activities and the people around them, as captured in the following excerpts:

‘Sometimes, my emotions will be mixed up, my emotions will be so so terrible such as being too moody that I would not even get out of my room, I cannot cope at all nowadays.’ (Adolescent 1, female, MHI)

‘She is getting worse, she cannot cope, she is always collapsing, is it obvious that nothing is working out fine? Her mood keeps changing like the depression is coming on her most times.’ (Parent 1, female, MHI)

‘It is worrying me a lot because I am a fun person. I love laughing a lot and making the people around very happy but all those things changed because I cannot cope with this condition and it is very discouraging. It is really so strange to me because most times, I feel powerless, I cannot handle.’ (Adolescent 2, female, MHU)

According to the Diagnostic and Statistical Manual of Mental Disorders (American Psychiatric Association 2013:155), depression in adolescents is associated with sadness, feelings of hopelessness and moodiness, thus making it difficult for the predisposed individual to cope in a society. Khesht-Masjedi et al. (2017:775) posited that adolescents with depression also complain of restlessness, irritability, anger and sadness; however, these complaints can vary amongst sexes. Roohafza et al. (2014:944) argued that most coping difficulties encountered by adolescents with depression are because of the excessive and pathological effects of the depressive condition in adolescents. The coping mechanisms of such adolescents can be further compounded by the perceived poor understanding of their challenges by parents, siblings and peers, thus lacking the capability to support them (De Mol et al. 2018:3). Mondimore and Kelly (2015:1–2) stated that depression amongst adolescents poses huge stress on many parents because of treatments required by the adolescent. Parents can also be traumatised by the thoughts of the treatment and general negative impacts of the condition on the family. Furthermore, Huang et al. (2014:256) argued that depression in adolescents is a mediator of parental stress, necessitating the examination of the parental–adolescent relationship for good recovery outcomes.

**Negative attitudes from the support systems:** Some adolescents and their parents complained of negative treatment from support systems. Poor understanding of depression and management outcomes by parents negatively contributed towards worsening of the condition of children. Support systems in this context include families, relatives and mental healthcare practitioners. With regard to the negative experiences, participants interviewed in the study indicated as follows:

‘Some of the nurses are so rude neh, and some of them like I remember my first time of going to [*name of the hospital*] neh, so I saw this social worker neh, she was so rude to me telling me how like she was saying I am a mama’s baby instead of motivating me.’ (Adolescent 17, female, MHU)

‘I usually fight with my child, you know these kids can be stubborn, you tell her to do this, she will not do it, so we fight. Her elder sister is not usually around, so am with this one, so we fight a lot, we hardly talk, which was really making her depression worse but I now see that I am not doing enough.’ (Parent 8, female, MHU)

‘No one understands me; they are not concerned about my emotional health. My dad is not listening to me, he does not care how I feel, it makes things difficult for me, I feel lonely, I have tried killing myself many times. My home is not favouring my emotional health; it hurts me a lot.’ (Adolescent 8, female, MHI)

‘I do not just understand how they operated because I think they were against her like they were not treating her fine; they were disrespectful, because I used to tell them what was happening but they would not listen to me. It makes me feel so bad and sometimes, I will cry and eeeem what made me to cry was they were not listening to what I was telling them about my child[*‘s*] situation.’ (Parent 14, female, MHI)

Wang et al. (2020:8) posited that family plays an integral role in nurturing an adolescent. Hence, its dysfunction is a mediating factor to the development of low self-esteem, depression and poor management outcome for an adolescent with depression. Thus, members of the family should understand their place in the general development of children and the management of mental health conditions, such as depression. Similarly, Reardon et al. (2017:642) reported that negative attitude of mental health professionals towards parents and their mentally ill adolescents is a barrier to positive treatment. Hsiao, Lu and Tsai (2015:277) found that mental health nurses demonstrate negative attitudes towards people suffering from mental health conditions, such as depression. Participants in this study maintained that the negative attitude of mental healthcare practitioners constitutes a barrier to the positive management of depression amongst adolescents.

**Financial strain:** Adolescents and their parents revealed the mental health management of depression exposed them to financial constraints as captured in the following excerpts:

‘My problem is that I do not have money; I cannot manage financially because my child’s depression is costing me lots of money.’ (Parent 1, female, MHI)

‘We are poor, my mother is old. So, it is a life of struggle for us, the management is costing my mother the little grant she is getting from the government.’ (Adolescent 15, male, MHU)

‘I do not have the money because I kept spending the one I had on my child’s condition. I take her to consult at the hospital and that is much money from me. I am staying very far from the hospital and for me, that is not working; it is more challenging to me. I really need financial help.’ (Parent 2, female, MHU)

Mental health conditions and other chronic disease expose families to financial stress. Families with a child suffering from depression usually are subjected to financial burden because of the continuous visits to mental healthcare institutions (Zhao et al. 2019:1333). Magklara et al. (2015) maintained that depression leads to comorbid disorders on the adolescent, thus increasing financial constraints on families.

**School-related challenges:** Notwithstanding the mental health management of depression, some adolescents and their parents indicated that depression affects the educational life of the adolescent, coupled with its associated negative experiences as indicated in the following excerpts:

‘She told me that they bully her, her classmates, at school and that caused her problems like falling into depression more and more. So, I changed her from that school; I do not like it at all; I do not like it at all, that report really hurts me because no one wants the child to be bullied or not to feel loved you, know!’ (Parent 12, female, MHI)

‘I had to leave school because of the continuous episodes of depression.’ (Adolescent 17, female, MHU)

‘Things got worse and I had to leave school like things got worse because like eeem, I started getting more depressed and like I do not think the medical practitioners know what to do for me again.’ (Adolescent 17, female, MHU)

The above excerpts are consistent with scholarly reports by Dupéré et al. (2018:208) that depression amongst adolescents is significantly associated with school dropout. Chukwuere et al. (2020a:126) argued that depression amongst adolescents contributes to absenteeism, poor performance and dropout from schools. Furthermore, Jayashree et al. (2018:31) posited that depression amongst this age group is associated with poor performance and other adverse outcomes. Meanwhile, Banny et al. (2013:6) stated that bullying is one of the challenges faced by adolescents suffering from depression.

**Withdrawal:** Adolescents and their parents indicated that adolescents with depression withdraw from other people despite the management of their condition, which has devastating consequences that affect adolescents and people around them. Despite the mental health management being received by adolescents with depression, parents complained that the symptoms are not abating, as indicated in the following excerpts:

‘She was feeling withdrawn; she loves having her phone and headset always; she easily gets angry, if she comes back from school, she goes into her room as her younger sister told me. You know, I do go to work.’ (Parent 2, female, MHU)

‘She can lock herself up in the room for a long time and when she comes out, you will see her eyes red, showing she has been crying. When she is in her room, she does not like anyone to talk to her.’ (Parent 8, female, MHU)

‘Since I started having depression, I started staying on my own, I started avoiding my friends. When I come back from school, I love staying in my room alone maybe writing down how I am feeling or with my phone. I do not like talking to anyone.’ (Adolescent 11, male, MHI)

Morgan, Huebner and Ruffin (2019:1) stated that some adolescents suffering from depression tend to withdraw from their families and friends they normally interact with, which mostly represent the unpredictability of depression. Finning et al. (2019:929); Morgan, Shaw and Forbes (2013:1118) maintained that social withdrawal is one of the features of depression in adolescents.

#### Theme 2: Methods of managing depression in adolescents

Despite the negative experiences of parents and adolescents, participants proposed the following management approaches and strategies to mitigate symptoms of depression as discussed in the below subsection.

**Provision of a therapeutic environment in hospitals:** Therapeutic environment is crucial in providing adolescents with depression and their parents with the needed sense of caring presence and acceptability crucial for facilitating adolescent recovery. Thus, adolescents and their parents highlighted the need for a therapeutic environment in hospitals to cater for young people suffering from depression as captured in the following excerpts:

‘To me, I think the hospital should help us by being friendly to us like the nurses and doctors; they should try to understand what we are passing through emotionally. At least, when we come with our problem, we should be smiling while going home.’ (Adolescent 16, male, MHI)

‘Like the hospital should be friendly, it must help us to heal. I mean this environment is not friendly to us like the environment is not friendly like is just more depressing so I feel they should introduce things that would make us feel better.’ (Adolescent 17, female, MHU)

‘Again, you see this hospital should be more therapeutic friendly starting from the mental health officers down to the cleaners. They should not be hostile because this depression is a thing of emotion so if the environment is therapeutic friendly, these children will be loving to come for treatment, with that, their health can improve.’ (Parent 9, female, MHI)

A therapeutic environment facilitates adequate patient–nurse relationship. According to Kornhaber et al. (2016:537), the nurse–patient relationship is healthy for the development of positive clinician–patient experience. Cousin et al. (2012:193) further maintained that therapeutic relation emerges from a conducive environment filled with genuine interest, friendliness, empathy, and capable of providing the patients with the necessary support for recovery.

**Continuous monitoring by mental healthcare practitioners:** Most parents indicated the need for continuous monitoring of their children by all mental healthcare practitioners in mental healthcare institutions and units, as captured in the following excerpts:

‘I see some progress with consulting the psychologist; they are monitoring her condition well. The psychologist kept telling me that she will be fine. I see some progress with the consultation.’ (Parent 1, female, MHI)

‘She has been seeing a doctor and a psychologist on a regular basis. So, they are monitoring her very well with the necessary help, such as giving her drugs and counselling, even with that, I do not still understand her. It was the psychologist who told me about supporting her emotionally.’ (Parent 8, female, MHU)

‘The first thing eeee, she has been seeing different psychologists, social workers, nurses, eeeem, and eeem. I was not ok with the way they handled the situation, like there were some of the mental health personnel who were just fair to her like talking to her nicely.’ (Parent 14, female, MHI)

Despite the differences in views as indicated above, the literature shows that psychological therapies, in combination with antidepressants, are used in the management of depression in adolescents (Cox et al. 2014:2). Costello et al. (2019:2) posited that counselling is amongst the strategies used in managing depression, which usually takes place after the initial diagnosis of the condition. The counselling section helps to enlighten the adolescents with depression on the available option for managing depression, as well as other treatment plans that could assist in mitigating symptoms. Isa et al. (2018:11) argued that pharmacotherapy is widely used in managing depression amongst adolescents in some African countries, as the first line of treatment, which can be attributed to lack of local evidence regarding the effectiveness of psychological intervention in these regions. Gabbay et al. (2018:2) added that despite the effectiveness of medication in the treatment of depression, it does not work on many adolescents with depression, whilst some adolescents tend to reject medication as their treatment option.

**Positive attitudes from support systems:** Adolescents and their parents highlighted the need for positive attitudes from support systems, such as families, nurses and the whole community. Support systems can be internal or external, an indication that such support could come from loved ones, such as friends, teachers, social groups and the community. Adequate support systems help adolescents with depression in their feeling of belonging to the society, as indicated in the following excerpts:

‘I feel my mother understands me now. I definitely feel so because she told me that she read what I have written and realised that she was not listening to me enough. All she thought was that I am faking everything, especially when I self-harmed.’ (Adolescent 1, female, MHI)

‘Families should do more in helping their children emotionally, supporting them, besides providing material things for them, emotional support is very necessary.’ (Parent 2, female, MHU)

‘I will suggest support groups in the community for the children where they can share their challenges and be not judged and getting emotional support.’ (Parent 2, female, MHU)

Families, as an internal support system, play an important role in the recovery of adolescents diagnosed with depression (Roohafza et al. 2014:946). Positive support systems reduce depressive symptoms in adolescents and foster their coping mechanisms (Van Harmelen et al. 2017:2312). Families can establish appropriate communication with their children regarding mental health conditions and help them in building their individual resilience and proper coping skills (Beardslee, Chien & Bell 2011:248). In addition, supportive environments constitute a reliable support system for mitigating depressive symptoms amongst adolescents (Dardas 2019:251). Roohafza et al. (2014:947) posited that perceived support systems, especially positive re-interpretation, are crucial in recovery of adolescents from depression. Bean et al. (2019:52) stated that inadequate social support negatively affects the recovery process of the adolescents with depression. Van Droogenbroeck, Spruyt and Keppens (2018:5) further add that inadequate social support generally escalates other mental health problems amongst adolescents with depression.

**Use of media to teach people about depression:** Management of depression amongst adolescents is very important, thus there is the need for appropriate measures to foster recovery and enable adolescents’ lead better life. Media houses have a wide-range capacity to disseminate information, hence an appropriate avenue for promoting mental health-related information that can assist in recovery of children from depression, as captured in the following excerpts by participants:

‘I think there should be more campaigns about this depression because it is really a big issue. Since it is about adolescents, television houses should even make cartoons about depression, display them on televisions so that children can be watching in order to know more about depression at such young age and also know how to handle difficulties.’ (Parent 7, female, MHI)

‘Probably, they can use radio in educating people because like most people listen to radio or they could use I do not know, they should go out like any possible measure like radio or television, something like that.’ (Adolescent 1, female, MHI)

These views are in line with the findings of Keles, McCrae and Grealish (2020:79) that media platforms present an important avenue for the dissemination of information about depression in struggling adolescents through awareness campaigns, social interaction or support groups. Such platforms enhance help-seeking behaviours amongst adolescents and provide them with appropriate management strategies to deal with their condition.

## Conclusion

This study focused on the experiences of adolescents and their parents on the mental health management being received by adolescents with depression in the NWP, South Africa. The findings highlighted the experiences of adolescents suffering from depression, the plight of parents taking care of them and crucial information on the management of depression, which could facilitate recovery of adolescence from depression amongst this age group.

## Limitations of the study

This study followed a qualitative research approach, and hence, the findings cannot be generalised for the entire province of South Africa, and rather a similar study can be replicated in other provinces. The researcher employed the service of a translator during the interview session with one of the parents, and hence, may have failed to capture all the vital information crucial for the study. Furthermore, the researcher took longer than expected to recruit adolescents and their parents.

## Recommendations

Based on the findings of the study, there is a need for mental healthcare practitioners to work towards improving the experiences of parents and their adolescents receiving management for depression. It is also recommended that similar studies be conducted in other provinces of South Africa in order to have a clear picture of the experiences of parents and their adolescents suffering from depression regarding their mental health management, which may foster proactive measures that can make life better for adolescents and their families. There is also a need for mental health institutions and units to put in place measures for active follow-up care for adolescents suffering from depression. The study provides an account of the experiences of adolescents and their parents, which can be shared with mental healthcare practitioners so that they can reflect on how to plan their mental healthcare services to acknowledge and address these needs of the adolescents and parents. Their experiences will always be challenging because depression is a challenging disorder; however, having this insight can help mental healthcare practitioners to prepare better for future challenges.
